# Time-dependent, patient-centered perceptions of quality measures for total joint arthroplasty: a cross-sectional, choice modeling study

**DOI:** 10.1186/s12891-025-08284-w

**Published:** 2025-01-13

**Authors:** Jacie L. Lemos, Jessica M. Welch, Derek F. Amanatullah, Lauren M. Shapiro, Alex H. S. Harris, Robin N. Kamal

**Affiliations:** 1 Department of Orthopaedic Surgery, University of Michigan, Ann Arbor, MI USA; 2 Department of Orthopaedic Surgery, Duke University, Durham, NC USA; 3 VOICES Health Policy Research Center, Department of Orthopaedic Surgery, Stanford University, 450 Broadway Street MC: 6342, Redwood City, CA 94603 USA; 4 UCSF Department of Orthopaedic Surgery, San Francisco, CA USA; 5 Department of Surgery, VA Palo Alto Healthcare System Stanford University, Palo Alto, CA USA

**Keywords:** Total joint arthroplasty, Value-based care, Quality definitions, Patient reported outcome measures, Patient-centered care

## Abstract

**Background:**

As value-based care arrangements continue to assess quality of care and costs, comprehensive and patient-centered definitions of quality of care are required. While patient-reported outcome measures are increasingly integrated into quality assessments following total joint arthroplasty (TJA), patient perceptions of quality paired with the phase of surgical care has not been described. The purpose of this study was to assess how TJA patients perceive measures of quality of care and assess if these perceptions change based on the phase of care.

**Methods:**

Patients who had undergone a TJA within the past two years or had a scheduled TJA within the next 6 months completed a questionnaire designed using best-worst scaling, a method used to measure individuals’ priorities by asking participants to make repeated selections of the best and worst items in a series of subsets of items. Subanalyses were calculated to compare each phase of care (preoperative, short term postoperative, and long term postoperative).

**Results:**

A total of 153 patients completed the questionnaire; 36 were preoperative, 55 were short term postoperative, and 62 were long term postoperative. Patients placed the highest value on improving activities of daily living (β = 1.03, CI = 0.90-1.16), decreasing pain (β = 0.65, CI = 0.53-0.76), and avoiding re-intervention (β = 0.64, CI = 0.52-0.76). Decreasing pain ranked as a higher priority preoperatively compared to short term postoperatively, and subsequently increased in priority again after 6 months. Avoiding reintervention was less important to patients preoperatively compared to postoperatively. Avoiding complications was more important to patients preoperatively compared to postoperatively.

**Conclusions:**

Matching outcome assessments with how patients assess their quality of care throughout the TJA recovery process can inform phase-specific quality improvement initiatives and value definitions. Activities of daily living should be measured across phases of care and into long-term recovery. TJA value dashboards should align with these patient-driven perceptions of quality.

**Level of evidence:**

Level III, cohort study.

**Supplementary Information:**

The online version contains supplementary material available at 10.1186/s12891-025-08284-w.

## Background

Total joint arthroplasties (TJA) are increasingly common procedures as the population ages. Total hip arthroplasties (THA) and total knee arthroplasties (TKA) are projected to increase by 171% and 185% from 2014 to 2030 in the US, respectively [1]. As a result, total Medicare costs for TJA procedures are expected to rise to approximately $50 billion by 2030 [2]. Outcome measure sets assessing quality of care after TJA have traditionally measured objective outcomes, such as postoperative complications. For example, Medicare has measured hospital length of stay, 30-day mortality rate, 30-day complication rate, and 30-day readmission rate [3, 4]. More recently, care delivery systems and payers have placed greater value on aspects of quality care from the patient perspective, such as utilizing patient reported experience measures (PREMs) and patient reported outcome measures (PROMs) [5]. Patient reported measures such as the Press Ganey Patient Experience survey [6], Knee injury and Osteoarthritis Outcome Score - Joint Replacement (KOOS-JR) and the Hip disability and Osteoarthritis Outcome Score - Joint Replacement (HOOS-JR) [7] are being used to define quality after TJA [8].

Value dashboards have been developed that use a combination of objective outcomes (e.g. infection rates) and patient-reported measures (e.g. HOOS-JR, KOOS-JR) to assess value of care [9]. These value portfolios or dashboards are limited in that they often do not include multiple stakeholders (i.e. patients) in the selection of outcome measures and assume that each measure reported by the patient is equally important (and weighted) along all phases of care. It is possible that patients measure their quality of care differently based on which phase of care they are in. A study of fracture patients found that patients had changing priorities (i.e. work-related recovery vs. physical recovery) based on the period of recovery that they were in [10]. The purpose of this study was to assess how TJA patients perceive measures of quality of care and assess if perceptions of quality of care change based on the phase of care. These data can guide the use of patient-centered outcome measures to comprehensively define quality for TJA.

## Methods

### Study design and setting

This cross-sectional, choice modeling study was conducted with approval from the Stanford institutional review board. Overall, we elicited patient preferences for TJA outcomes at multiple time points using terminology from existing patient-centered and physician-derived measures. This generated a rank-ordered list of TJA quality of care measures that demonstrate relative patient preferences, overall and at three specific time points. This cross-sectional study was created with adherence to the guidelines of the Strengthening the Reporting of Observational Studies in Epidemiology (STROBE) Statement Checklist.

**Fig. 1:**
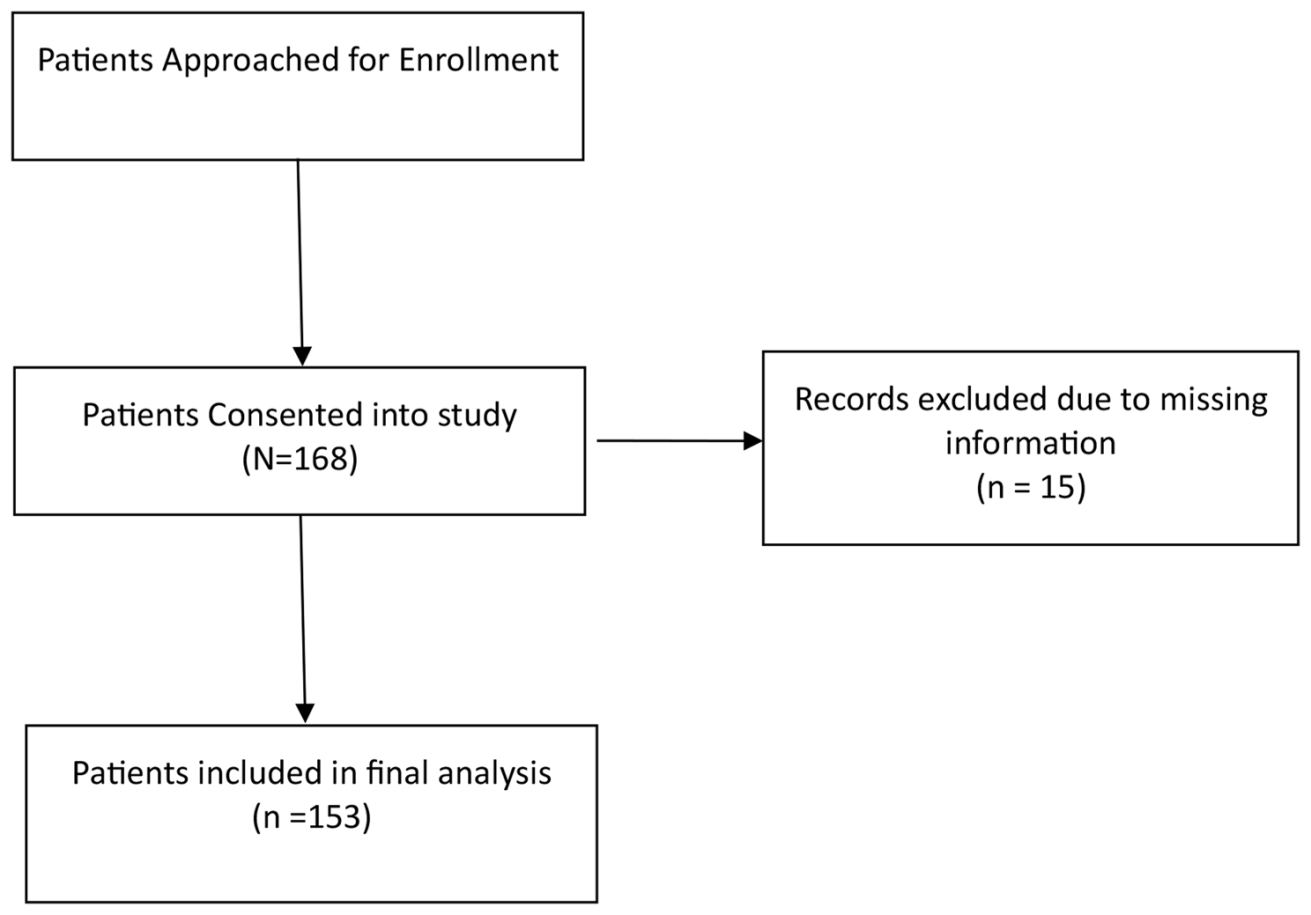
Enrollment flow chart

We recruited patients presenting to a total-joint replacement clinic, including pre-operative visits and post-operative follow-up visits (Fig. 1). Participants were eligible if they (1) underwent TKA or THA within two years or (2) had a scheduled TJA within the next 6 months. A trained member of the research team screened for eligibility using this criterion and approached eligible patients for consent. Eligible, consented participants completed a demographic questionnaire, followed by a 13-item questionnaire to measure patient preferences. Participants were excluded if they were under the age of 18 or did not speak English. Participants who submitted the questionnaire but failed to fill out all required questions were excluded. Questions regarding gender, educational status, and household income were optional. Written, informed consent was obtained from all participants.

### Development of outcome measures questionnaire

**Fig. 2:**
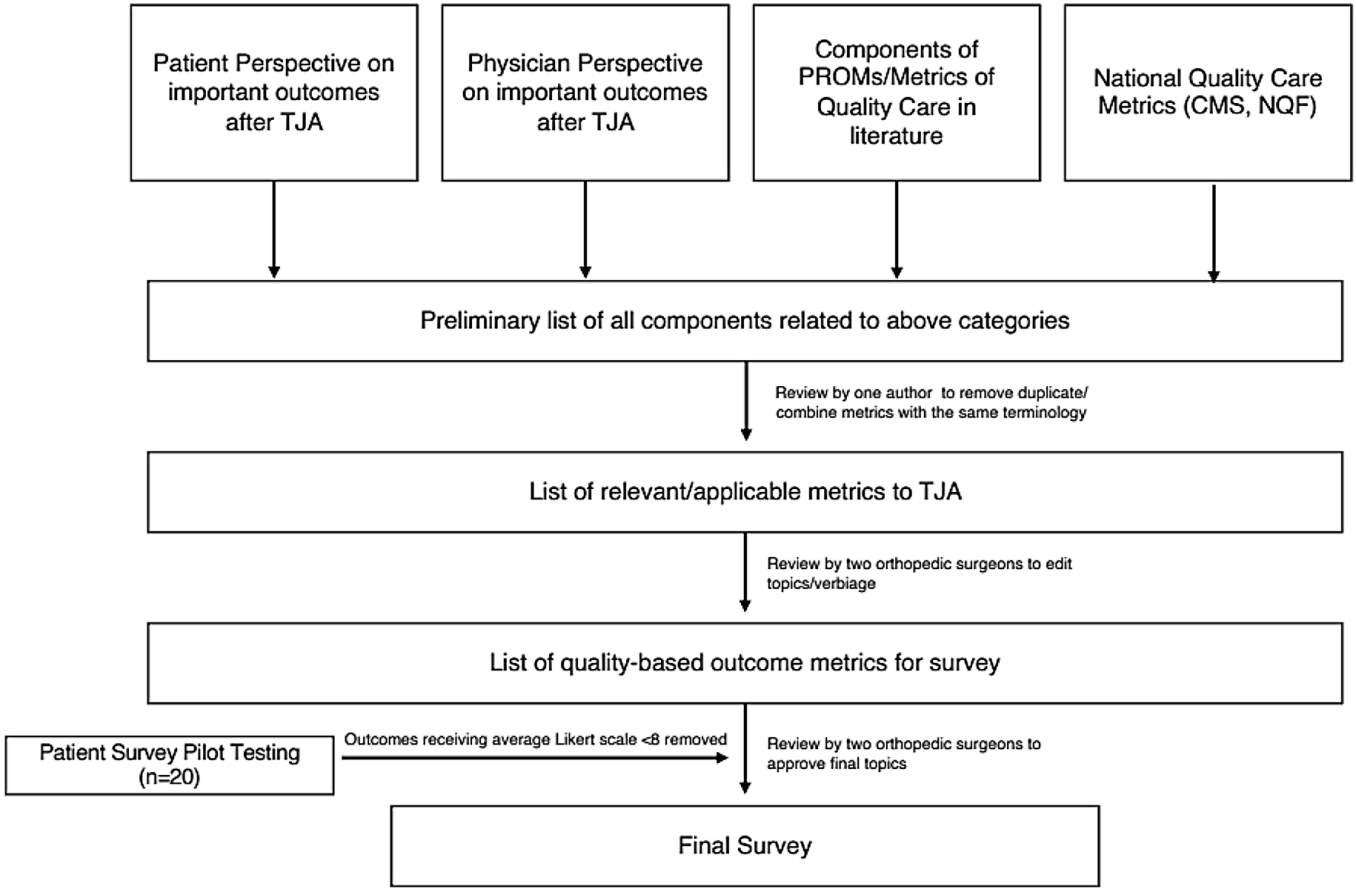
Steps of outcome generation process

To generate a choice modeling questionnaire, we conducted multi-step identification, iteration, and refinement of questionnaires (Fig. 2). We reviewed prior publications that define outcome measurements in TJA (from both physician and patient perspectives), outcome metrics commonly used in clinical settings (i.e. H/KOOS-JR), national quality metrics (i.e. Centers for Medicare & Medicaid Services – CMS, National Quality Forum – NQF), and frameworks of quality of care that address outcomes (i.e. Porter’s Outcome Measure Hierarchy) to compile a list of outcomes relevant to TJA (e.g. relief of pain, ability to return to specific activities, etc.). Each outcome with a measurable time to achievement (i.e. return to specific activities) included a time factor (i.e. “quick return to activities” vs. “return to activities”).

Two orthopedic surgeons (RNK and DFA) iteratively revised this preliminary list to ensure all components were written in nontechnical language and were relevant to TJA procedures. For example, measures of mortality rates were removed because it is assumed that this is a top priority for all patients and mortality risk is low in TJA. To evaluate response variability we first prepared a questionnaire that asked patients to rate the importance of the 19 outcomes identified using a Likert scale (1–10). Likert scales often generate a ceiling effect in which participants tend to score items at the maximum value offered. However, in this case the Likert scale method allowed for identification of items which were least important to participants (Supplementary Table 1). 20 pilot patients completed the preliminary questionnaire and a ceiling effect was observed (as expected), with several patients ranking 10 for all items on the list. A ranking of 8 or higher was identified as an appropriate cutoff value as a notable disparity was identified between outcomes rated 8 or above and those rated below 8. Therefore outcomes receiving an average score of less than 8 were removed from the list for the final questionnaire, leaving a list of 13 outcomes (Table 1).

**Table 1:** Summary & definitions of best worst scaling outcomes

Choice item	Description
Activities of Daily Living (ADL)	Improved motion and daily function (ex. walking up and down stairs, getting out of a chair, etc.).
Pain	Relief of pain.
Reintervention	Avoiding needing a repeat surgery in the future.
Complication	Avoiding complications (ex. infection, readmission, etc.).
Quick Activities of Daily Living (qADL)	*Quick* improvement in motion and daily function (ex. walking up and down stairs, getting out of a chair, etc.)
Hobby	Improvement in ability to participate in specific activities such as hobbies, sports, etc.
Quick Pain (qPain)	*Quick* resolution of pain.
Quick Hobby (qHobby)	*Quick* improvement in ability to participate in specific activities such as hobbies, sports, etc.
Communication	Access to open, frequent communication with care team/ caregivers.
Knowledge	Gaining knowledge and feeling informed about process of surgical care.
Heard	Feeling heard and respected by care team/ caregivers.
Positive	Increased positive emotions (ex. confidence and peace of mind)
Negative	Minimized negative emotions (ex. fear, uncertainty, sadness)

### Best-worst scaling (BWS) method

We next designed and conducted a preference elicitation questionnaire based on the established outcomes and the best-worst scaling (BWS). BWS is a method used to measure individuals’ priorities by asking participants to make repeated selections of the best and worst items in a series of subsets of items [11]. By utilizing BWS case 1 design, also known as object case, patients were asked to assess each outcome attribute as an individual concept. This design measures the relative importance among choice alternatives in a way that is easy for participants and overcomes common challenges in survey research (e.g. ceiling effects and high cognitive burden). It generates a ranked list of items that may be difficult to elicit from participants with other survey methods (and the biases that come with them) [11]. This ranking approach has been used in healthcare to determine patient preferences such as what factors influence patient choice of doctor [12], factors of treatment that encourage seeking care [13], and assessing treatment options [14].

To ensure validity of the BWS, we utilized a balanced incomplete block design which ensures that all choice sets are equal in size and all choice options appear equally often and co-appear equally often with each other choice option [15]. Our questionnaire included v = 13 outcome measures/choice items, b = 13 choice sets/question blocks, k = 4 items per set, and *r* = 4 repetitions of each choice item. Participants were asked to select which of the 4 outcome attributes were most and least important to them regarding their recovery from TJA. A sample question is included in Supplementary Fig. 1.

### Sample size

The ideal method for the determination of sample size for discrete choice analysis is unclear, with few taking into account the desired precision [16]. We have therefore used an opportunistic sample size based on what was reasonable in the projected time frame, and consistent with the recommendations of prior literature. Recommendations suggest that sample sizes over 100 participants provide reliable modeling of preference data [11, 17]. Previous BWS investigations of preferences in healthcare settings (with a similar number of survey items) have used similar sample sizes [18–21].

### Statistical analysis

**Fig. 3:**
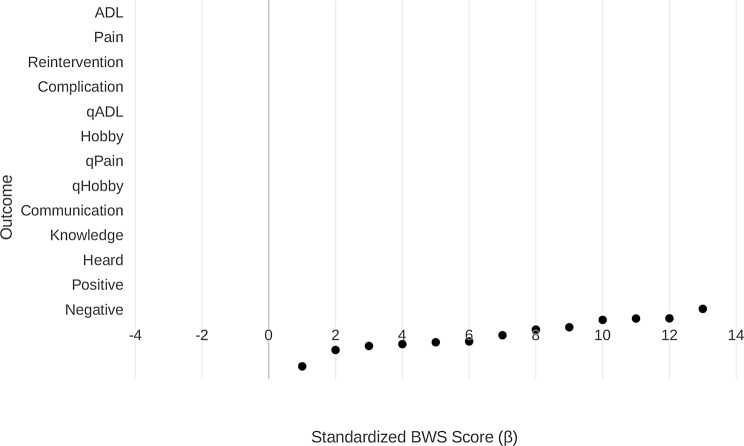
Standardized BWS scores (β coefficients) plotted for each of the 13 outcomes with standard error bars. ADL = activities of daily living; qADL = quick return to ADL; qPain = quick resolution of pain; qHoppy = quick return to hobbies

From participant responses we calculated individual BWS scores for each of the 13 choice items, defined as number of times each outcome was chosen as the most and least important. We used RStudio and Microsoft Excel to complete our analysis. Our analysis assigned the most important item a score of + 1, the least important item a score of -1, and all other items 0 per choice set. We then used a multinomial logit model (using R programming) to create a ranked list based on BWS scoring. The multinomial logit model summed BWS for each item, divided that value by the total availability of the choice item (4 times per questionnaire * number of participants), and created a ranked order based on the results. Specifically, the model produced the standardized score (β value), the standard error, and the 95% confidence interval [22]. These were then plotted to demonstrate the relative importance of each item (Fig. 3). The standardized score of each item represents the relative strength of importance, or salience, of each item across the entire sample (i.e. higher value indicates increased importance). To compare outcome preferences across recovery timepoint, we repeated the multinomial logit model by subgroup (recovery timepoint).

## Results

One hundred and sixty-eight patients completed the questionnaire. Fifteen patient responses were excluded from analysis due to missing information. 153 patients were included in the final analysis, with a mean (± standard deviation) age of 66 ± 11 years. Of these, 91 (59%) were female, 36 (24%) were preoperative, 55 (36%) had undergone surgery within 6 months (short term postoperative), and 62 (41%) had undergone surgery more than 6 months prior (long term postoperative) (Table 2). The patients excluded due to missing information had similar demographic characteristics compared to the included patients with 7 (47%) being female and a mean (± standard deviation) age of 68 ± 14 years.

**Table 2:** Patient demographics

Patient characteristic		Number of patients, *n* (%)			
		Total (*n* = 153)	Pre-Op (*n* = 36)	Short-term post-op (*n* = 55)	Long-term post-op (*n* = 62)
Age (mean + SD)		66 ± 11	66 ± 11	65 ± 12	67 ± 10
Gender					
	Male	62 (41%)	15 (42%)	21 (38%)	26 (42%)
	Female	91 (59%)	21 (58%)	34 (62%)	36 (58%)
	Other/Prefer not to say	0 (0%)	0 (0%)	0 (0%)	0 (0%)
Race*					
	White	101 (66%)	23 (64%)	32 (58%)	46 (74%)
	African American	12 (8%)	3 (8%)	5 (9%)	4 (6%)
	Hispanic	17 (11%)	2 (6%)	11 (20%)	5 (8%)
	Asian	15 (10%)	5 (14%)	5 (9%)	5 (8%)
	American Indian or Alaska Native	1 (1%)	0 (0%)	1 (2%)	0 (0%)
	Other	9 (6%)	3 (8%)	4 (7%)	2 (3%)
Employment Status					
	Working	35 (23%)	11 (31%)	11 (20%)	13 (21%)
	Retired	74 (48%)	17 (47%)	24 (44%)	33 (53%)
	Disabled	28 (18%)	6 (17%)	13 (24%)	9 (15%)
	Unemployed	9 (6%)	1 (3%)	4 (7%)	4 (6%)
	Other	7 (5%)	1 (3%)	3 (5%)	3 (5%)
Educational Status					
	Some High School	7 (5%)	0 (0%)	4 (7%)	3 (5%)
	High School Graduate	22 (14%)	5 (14%)	13 (24%)	4 (6%)
	Technical/Trade School	11 (7%)	2 (6%)	4 (7%)	5 (8%)
	Some College	40 (26%)	10 (28%)	18 (33%)	12 (19%)
	4 Year College	39 (25%)	13 (36%)	8 (15%)	18 (29%)
	Advanced Degree	31 (20%)	6 (17%)	8 (15%)	17 (27%)
	DNR	3 (2%)	0 (0%)	0 (0%)	3 (5%)
Insurance*					
	Medicaid/Medi-Cal	44 (29%)	11 (31%)	11 (20%)	22 (35%)
	Medicare	81 (53%)	16 (44%)	29 (53%)	37 (60%)
	Private	45 (29%)	10 (28%)	19 (35%)	15 (24%)
	Supplemental	39 (25%)	10 (28%)	14 (25%)	14 (23%)
	Other	13 (8%)	1 (3%)	10 (18%)	3 (5%)
Household Income					
	<$50,000	59 (39%)	15 (42%)	21 (38%)	23 (37%)
	$50,000–99,999	24 (16%)	4 (11%)	12 (22%)	8 (13%)
	$100,000-149,999	33 (22%)	8 (22%)	12 (22%)	13 (21%)
	$150,000-199,999	16 (10%)	4 (11%)	4 (7%)	8 (13%)
	>$200,000	15 (10%)	5 (14%)	3 (5%)	7 (11%)
	DNR	6 (4%)	0 (0%)	3 (5%)	3 (5%)

*Patients were allowed to select multiple racial identities and insurances. (DNR = did not report)

The outcome development process yielded 13 outcomes for the final questionnaire. Overall, patients placed the highest ranking on improving activities of daily living (ADLs) (β = 1.03, CI = 0.90–1.16), followed by reducing pain (β = 0.65, CI = 0.53–0.76), avoiding reintervention (β = 0.64, CI = 0.52–0.76), and avoiding complications (β = 0.58, CI = 0.47–0.70) (Fig. 3). Reducing negative emotions had the lowest BWS standardized score (β=-1.29, CI=-1.43- -1.15). Patients ranked improving ADLs generally above improving ADLs quickly (β = 1.03, CI = 0.90–1.16 versus β = 0.29, CI = 0.14–0.40), and ranked reducing pain generally above reducing pain quickly (β = 0.65, CI = 0.53–0.76) versus β=-0.04, CI=-0.15- 0.08).

**Fig. 4:**
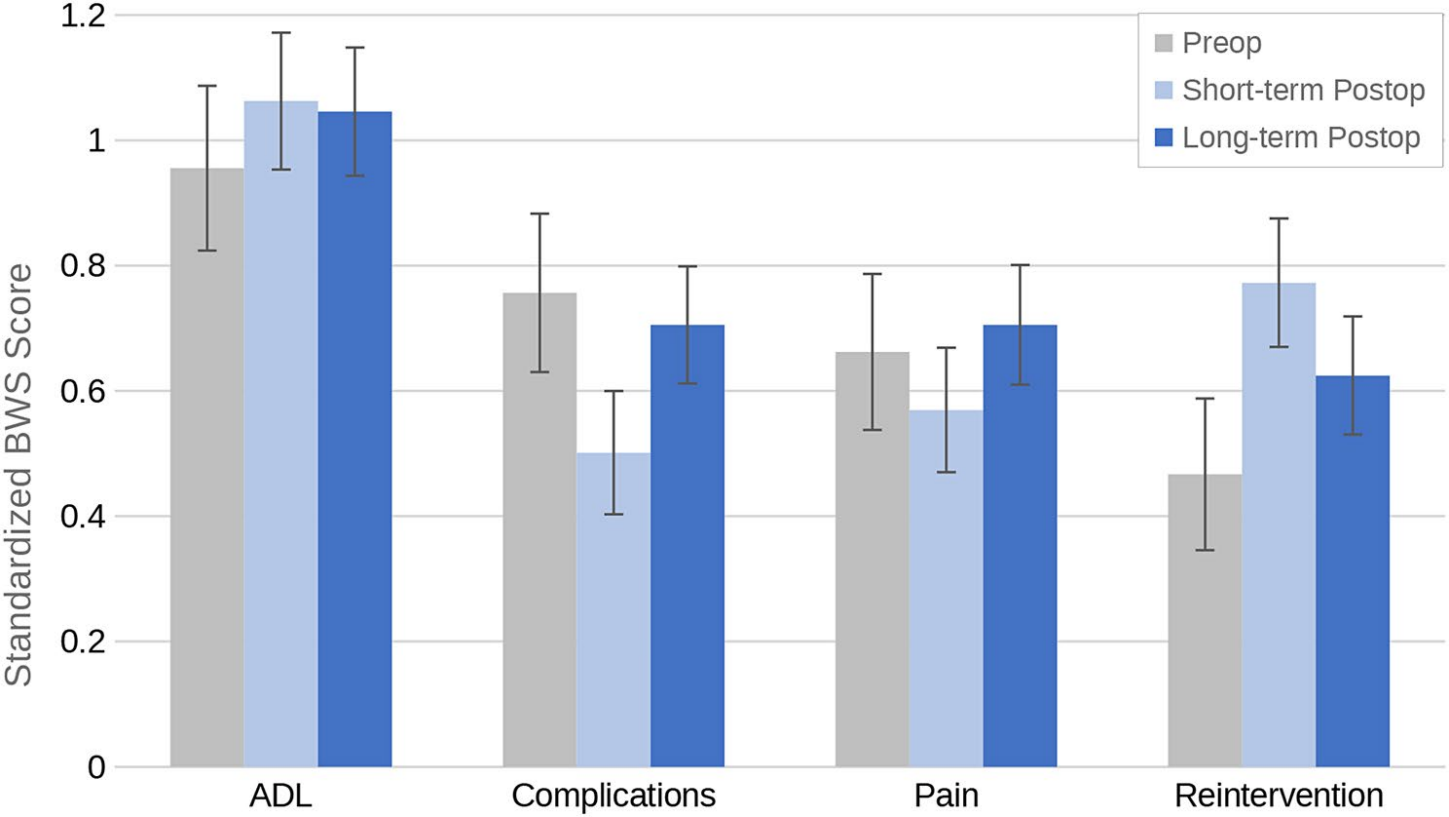
Top 4 ranked items from the regression model by timepoint

Improving ADLs was consistently ranked as the highest priority for quality care across all three time points (β = 0.96, 1.06, and 1.05, respectively, Fig. 4, Supplementary Table 2). Avoiding reintervention was ranked more important to patients postoperatively as compared to preoperatively (β = 0.47, 0.77, and 0.62, respectively, Supplementary Table 2). Pain was ranked more important to patients preoperatively as compared to within 6 months after surgery (β = 0.66 versus 0.57, respectively, Supplementary Table 2), but increased in priority again after 6 months (β = 0.71). Avoiding complications was ranked more important to patients preoperatively as compared to postoperatively (β of 0.76, 0.50, and 0.56, respectively, Supplementary Table 2). While there was variation in patient preferences across the timepoints, the top four outcomes consistently were improving ADLs, avoiding complication, reducing pain, and avoiding reintervention.

## Discussion

In this study, we elicited patient perceptions for TJA quality of care overall and across timepoints during the recovery process. Current value calculations often assume that each measure reported by the patient is equally important (i.e. weighted) over time, and there is no standardized framework for collecting these measures across all phases of care [9, 23]. We therefore demonstrate an argument for time-based outcome measures when assessing quality of care. Overall, patients prioritized improving ADLs, reducing pain, avoiding reintervention, and avoiding complication. However, patient priorities shifted across the recovery process. Notably, reintervention became more important, whereas reducing pain and avoiding complications became less important.

While health care delivery systems have placed a greater emphasis on including patient perspectives in value definitions, the selection of which PROMs to use in value calculations for TJA has been largely unstructured. A systematic review of PROM instruments used following TKAs found 47 different instruments used, with only 6 following gold standard methodology [24]. The use of value calculations requires the consideration of what metric best estimates patient experience. For example, while a surgeon may prefer the rate of reoperation (a well-defined outcome) to drive the overall value of a procedure, we have found that patients more highly value functional improvement. These results demonstrate that patient perspective on value is dynamic and as the preoperative and immediate postoperative windows have passed, they redefine what is most important to them. This builds upon a growing body of literature demonstrating shifting patient perspectives throughout recovery. Lieberman et al. found that the majority of patients undergoing THA indicated a different list of reasons for undergoing arthroplasty preoperatively as compared to postoperatively [25]. Baker et al. found that while pre-operative patient-reported variables are increasingly being used to predict patient satisfaction after TKA, the most robust predictors of satisfaction were when post-operative and pre-operative patient-reported variables were considered together [26]. Our study shows that this shifting perspective impacts a patient’s definition of value.

These results indicate the need for longitudinal PROM collection. Short-term, single timepoint value dashboards neglect to incorporate the dynamic nature of value derived from TJA. For example, a value dashboard that only uses data from the immediately postoperative time point might overestimate value by claiming a patient’s pain has improved after surgery but fails to evaluate a patient’s ability to participate in their favorite physical activity a year later. Conversely, a single time-point value dashboard may underestimate value by demonstrating immediate postoperative pain, while failing to capture pain improvements following the development of pain management strategies and return to their favorite activity. Some of these shifting priorities will be patient-specific, however many of them will follow logical trends. For example, we found that re-intervention mattered most to patients in the short post-operative period, directly after they had undergone surgery. These trends can be used to more accurately calculate value in the context of shifting patient priorities. Additionally, in this study we found that TJA patients value overall improvements in ADLs and pain over quick improvements in ADLs and pain. Past literature indicates that TJA patients experiencing delays in pain and function postoperatively frequently report long term improvements in these categories [27–29]. Long-term PROM collection strategies for TJA are therefore needed.

The optimal timing of PROM collection following TJA has been debated [23, 30, 31]. For example, Canfield et al. recommended collection of PROMs only up until 6 months following TJA, reporting that peak improvements in function occur within the first 6 months [30]. However, the continued top ranking of ADLs across time points in our study indicates that improving ADLs is the top priority to TJA patients long after 6 months postoperatively, and at minimum remains a top priority up to two years postoperatively. A study assessing TJA recovery trajectories by Hesseling et al. showed that while the majority of patients demonstrated fast initial improvement at the 3 month mark, more than 12% of patients demonstrated little to no improvements at the 3 month mark (and subsequently improved or declined at the 12 month mark) [28]. Therefore a shorter follow-up may be missing key changes in a patient’s functional recovery. More research should be done to define the most optimal intervals for PROM administration following TJA, but our findings combined with prior work indicates a need for PROM collection at multiple time points, and in the long-term.

The results of this paper should be viewed in the context of several limitations. Our patients were recruited from a single clinic in the United States and represent a relatively homogenous patient population. Notably, we excluded non-English speaking patients, who may have differing outcome preferences than English speakers. As such, the findings discussed in this study should not be viewed as generalizable to all TJA patient populations. Additionally, we did not record the number of patients initially approached for the study and the demographic information of those who declined to participate, which prohibited us from assessing for selection bias. Discrete choice surveys measure stated preferences, which may differ from revealed preferences. Preferences may also be impacted by whether the patient’s procedure had complications, a covariate that was not assessed in this study. Additionally, the study measured patient preferences up to 2 years post-operatively and there is a possibility that preferences further change after this time. Finally, this study is also limited by its cross-sectional design – future work should follow one cohort across multiple timepoints to best control for extraneous factors.

Future research should evaluate how to incorporate weighted scoring systems into value estimations so that the outcomes most important to patients at a given time point are weighted more heavily. Further, TJA value assessments should be monitored for their follow-up time and incentivized to generate the most complete definition of value. For example, the CMS bundled payment program for TJA offers incentives for PROM reporting before and after TJA procedures [32]. Finally, future efforts to create patient-centered value definitions should also include other stakeholders in the care process such as insurance payers or caregivers.

## Conclusions

Improving ADLs was the most important outcome to TJA patients across recovery timepoints. Pain was more important to patients before surgery, while avoiding reintervention was more important to patients after surgery. Contrary to assumptions in the current use of PROMs for TJA, patients had evolving outcome priorities during different phases of care. As value-based care becomes a priority in TJA procedures, it is important that value assessments reflect this dynamic nature of patient definitions. Current efforts to define value for TJA propose surgeon-driven metrics and provide no gold standard for an optimal follow-up period. TJA value assessments should be patient-driven and should collect PROMs at multiple time points and over longer time periods.

## Electronic supplementary material

Below is the link to the electronic supplementary material.

Supplementary Material 1

## Data Availability

No datasets were generated or analysed during the current study.

## Abbreviations

TJA: Total joint arthroplasty
THA: Total hip arthroplasty
TKA: Total knee arthroplasty
PREM: Patient reported experience measure
PROM: Patient reported outcome measure
KOOS-JR: Knee injury and Osteoarthritis Outcome Score- Joint Replacement
HOOS-JR: Hip disability and Osteoarthritis Outcome Score - Joint Replacement
CMS: Centers for Medicare & Medicaid Services
NQF: National Quality Forum
BWS: Best-worst scaling
ADL: Activity of daily living
