# Supplementary material for: Time-dependent, patient-centered perceptions of quality measures for total joint arthroplasty: a cross-sectional, choice modeling study
Source: BMC Musculoskelet Disord. 2025 Jan 13;26:41. doi: 10.1186/s12891-025-08284-w (PMC11727673; doi:10.1186/s12891-025-08284-w)
Supplement: Supplementary file 1 — Supplementary Material 1 [file 12891_2025_8284_MOESM1_ESM.docx]

**Supplemental Digital Content**

**Supplementary Table I: Original List of 19 Outcomes by Patient-Reported Likert Score**


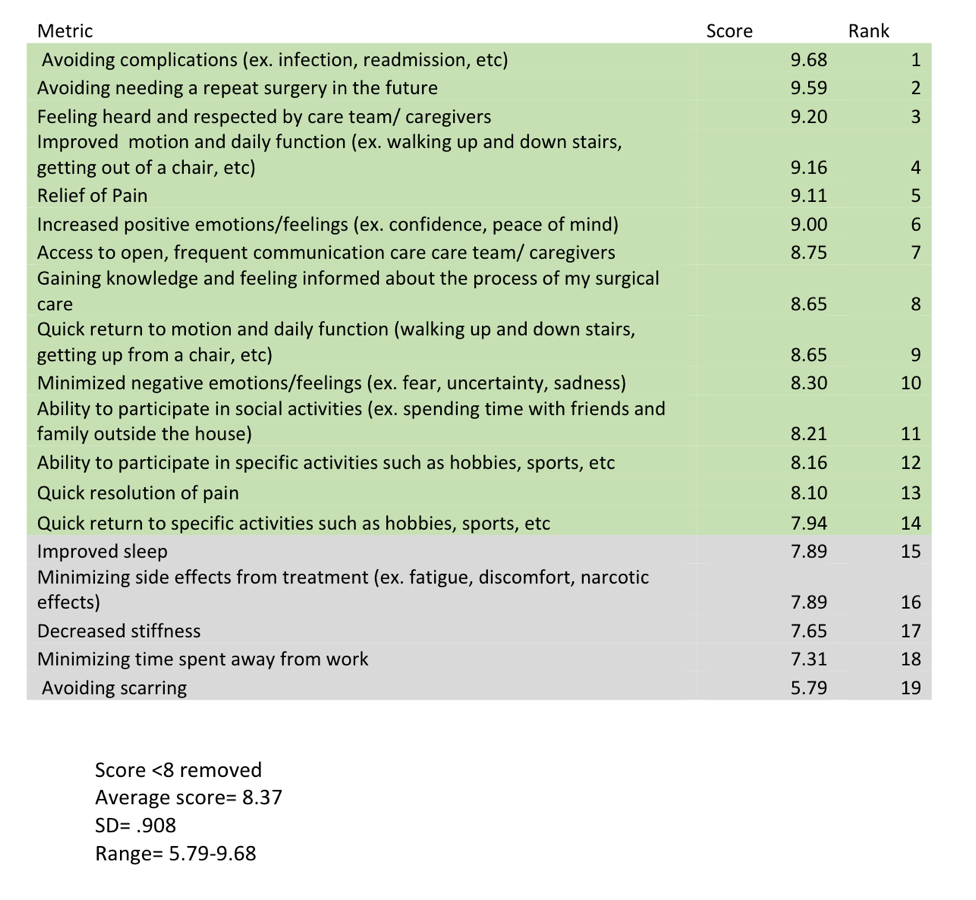


**Supplementary Table II: Best Worst Scoring Results**

| **Overall Rank** | **Choice Item** | **Best** | **Worst** | **Overall BWS Score (CI)** | **Preop BWS Score (CI)** | **Short-term postop BWS Score (CI)** | **Long-term postop BWS Score (CI)** |
| --- | --- | --- | --- | --- | --- | --- | --- |
| 1 | ADL | 321 | 31 | 1.03 (.90 - 1.16) | .96 (.70 -1.21) | 1.06 (.84 – 1.28) | 1.05 (.84 – 1.25) |
| 2 | Pain | 251 | 60 | .65 (.53 - .76) | .66 (.42 - .91) | .57 (.37 - .76) | .71 (.52 - .89) |
| 3 | Reintervention | 251 | 77 | .64 (.52 - .76) | .47 (.23 - .70) | .77 (.57 - .97) | .62 (.44 - .81) |
| 4 | Complication | 264 | 75 | .58 (.47 - .70) | .76 (.51 - 1.00) | .50 (.31 – .69) | .56 (.38 - .75) |
| 5 | Quick ADL | 60 | 201 | .29 (.14 - .40) | .29 (.06 - 53) | .30 (.11 - .49) | .27 (.09 - .45) |
| 6 | Hobby | 64 | 183 | .19 (.08 - .31) | .22 (-.01 - .46) | .12 (-.07 - .31) | .24 (.07 - .42) |
| 7 | Quick Pain | 64 | 251 | -.04 (-.15 - .08) | -.03 (-.26 - .20) | -.05 (-.23 - .14) | -.03 (-.21 - .14) |
| 8 | Quick Hobby | 71 | 168 | -.29 (-.40 - -.17) | -.24 (-.47 - .00) | -.39 (-.58 - -.20) | -.23 (-.40 - -.05) |
| 9 | Communication | 192 | 133 | -.32 (-.43 - -.21) | - .31 (-.54 - -.07) | -.28 (-.47 - -.09) | -.36 (-.54 - -.18) |
| 10 | Knowledge | 182 | 95 | -.39 (-.50 - -.28) | -.29 (-.53 - -.06) | -.41 (-.61 - -.22) | -.43 (-.61 - -.25) |
| 11 | Heard | 112 | 123 | -.47 (-.58 - -.35) | -.45 (-.69 - -.22) | - .57 (-.76- -.37) | -.39 (-.57 - -.21) |
| 12 | Positive | 115 | 202 | -.63 (-.75 - -.51) | -.62 (-.86 - - .374) | -.57 (-.76 - -.37) | -.70 (-.88 - -.51) |
| 13 | Negative | 36 | 384 | -1.29 (-1.43 - -1.15) | -1.58 (-1.89 - - 1.28) | -1.03 (-1.24 - -.82) | -1.39 (-1.61 - -1.17) |

**Supplementary Figure I: Sample BWS Survey Question**


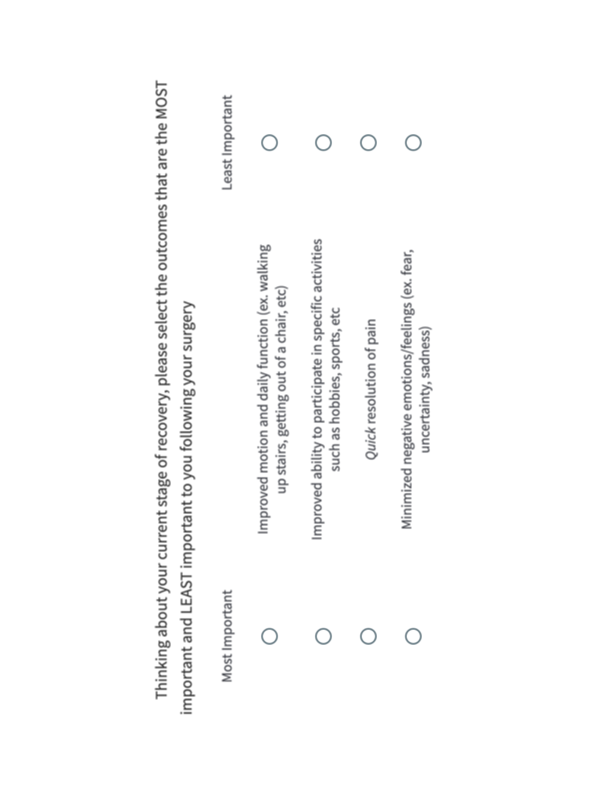


**Supplementary Figure II: Best Worst Scoring Results by Recovery Timepoint**
